# Measuring Software Timing Errors in the Presentation of Visual Stimuli in Cognitive Neuroscience Experiments

**DOI:** 10.1371/journal.pone.0085108

**Published:** 2014-01-07

**Authors:** Pablo Garaizar, Miguel A. Vadillo, Diego López-de-Ipiña, Helena Matute

**Affiliations:** 1 Deusto Institute of Technology, DeustoTech, Universidad de Deusto, Bilbao, Spain; 2 Cognitive, Perceptual and Brain Sciences, University College London, London, United Kingdom; 3 Faculty of Psychology and Education, Universidad de Deusto, Bilbao, Spain; Centre de Neuroscience Cognitive, France

## Abstract

Because of the features provided by an abundance of specialized experimental software packages, personal computers have become prominent and powerful tools in cognitive research. Most of these programs have mechanisms to control the precision and accuracy with which visual stimuli are presented as well as the response times. However, external factors, often related to the technology used to display the visual information, can have a noticeable impact on the actual performance and may be easily overlooked by researchers. The aim of this study is to measure the precision and accuracy of the timing mechanisms of some of the most popular software packages used in a typical laboratory scenario in order to assess whether presentation times configured by researchers do not differ from measured times more than what is expected due to the hardware limitations. Despite the apparent precision and accuracy of the results, important issues related to timing setups in the presentation of visual stimuli were found, and they should be taken into account by researchers in their experiments.

## Introduction

Since the days of Wilhelm Wundt, experimental psychologists have studied the temporal dynamics of the cognitive processes involved in perception and attention. Experimental apparatus that allow researchers to present visual stimuli for very short periods of time, such as tachistoscopes, quickly became popular in most experimental psychology laboratories. Since the 1980s, these complex and delicate devices have been replaced by personal computers and standard monitors, which are cheaper and easier to use. Moreover, experimental psychologists and cognitive neuroscientists now have several software alternatives that accurately present visual stimuli [1]. However, most of these tools run on standard personal computers (PCs), and this type of hardware is not optimized for the accurate presentation of visual stimuli. There are many limitations related to stimuli presentation durations caused by the underlying technologies of CRT and LCD displays and the timing mechanisms provided by non-real-time operating systems (e.g., Microsoft Windows on a PC). Both factors can have a significant impact on the accuracy and precision of the visual stimuli presentation, particularly in experimental paradigms that have very short Stimulus Onset Asynchrony (SOA) [2] [3] [4].

The goal of this research was to assess the potential discrepancies between the timing conditions defined by the researchers when using a subset of specialized software for the presentation of visual stimuli on common-use hardware and software (i.e., low-refresh-rate displays and non-real-time operating systems) and the actual onset and offset times of the visual stimuli detected using external photodetectors. Assessing the size of these potential timing errors is a first and necessary step in order to minimize and correct them. Whenever possible, we will also describe the way in which we were able to compensate these errors.

### CRT and LCD Displays

**Figure 1:**
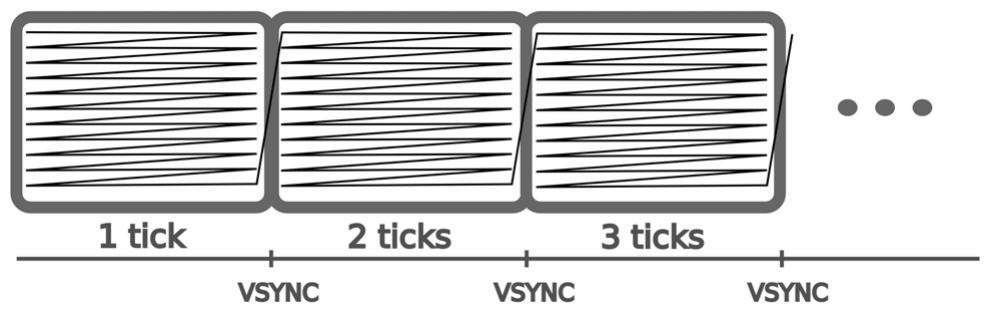
Representation of the path followed by the electron gun for a CRT monitor. At the end of each frame, the electron gun returns to the top left corner and starts again (VSYNC signal).

The widespread success of displays based on the cathode ray tube (CRT) established this type of monitor as the standard in computer-based experimental laboratories. A CRT monitor has an electron gun at the back that points to a glass screen covered with phosphors located at the front of the monitor. To display an image, the electron gun points to the top left corner of the screen and shoots a beam at the phosphors, briefly lighting them up. Once the image displaying process has started, the gun moves rapidly, and the beam energizes the entire screen, line by line down to the lower right corner. Once there, the electron gun turns off, points again to the top left corner, and the painting process starts again. The last step is controlled by the VSYNC (vertical synchronization) signal, and the refresh rate of the CRT monitor depends on how fast this signal is triggered (see Figure 1). The period between two VSYNC signals is known as a “tick,” and its duration depends on the refresh rate (e.g., 16.667 ms at 60 Hz and 10 ms at 100 Hz).

It is important to notice that the luminance of each dot of the image displayed by a CRT monitor is not constant but dependent on two factors: the refresh rate (i.e., the time the electron gun needs to energize it again) and the decay time, which depends on the phosphor type (1.5 to 6 ms for the frequently used P-22 phosphor). Therefore, researchers conducting experiments with CRT monitors should not assume a rectangular-shaped signal of the configured number of ticks of duration in their visual stimuli presentations, but a set of discrete pulses separated by the duration of a tick and the decay time.

Despite the continuous advances in liquid crystal display (LCD) technology, CRT monitors have been considered more suitable for the precise and accurate presentation of visual stimuli [5]. Their higher frame rates (typically around 85–100 Hz, but some models can achieve 240 Hz), greater number of frames per second (FPS), and shorter rise and decay times enable abrupt changes from one frame to another.

Nevertheless, because of their small size and low weight, LCDs are becoming more popular. Moreover, they offer some characteristics that increase visibility, such as the absence of flickering when displaying static images, and the poor temporal characteristics typically associated with LCD screens are based on studies of older LCD technology. Using current LCD displays, Lagroix, Yanko, and Spalek [6] found photometric estimates of the rise time that are far shorter (1–6 ms) than earlier estimates (20–150 ms) and approach those of CRTs (<1 ms). In addition, Wang and Nikolic [7] tested an inexpensive 120 Hz LCD display that was shown to have timing and stability characteristics at least as good as a CRT monitor. These findings suggest that LCD displays are suitable for studies that require high accuracy in the timing of visual presentations, but the truth is that both display technologies are affected by different limitations that make them inappropriate for brief stimuli presentations [8] [9].

The luminance of each dot of an LCD display is defined by the orientation of the liquid crystal molecules placed between two polarized glasses and two electrodes. To display an image, the array of electrodes modifies the amount of visible light emitted from the back of the display. Although this backlight is often assumed to be constant, its luminance level is pulse-width modulated. Instead of varying its intensity to adjust the luminance, it is switched off for brief periods of time (the higher the amplitude of the modulation, the lower the backlight luminance). The backlight modulation frequencies are far from the critical flicker frequency [10] and usually neglected, but might introduce undesirable effects in the visualization of stimuli (e.g., steady-state evoked visual potentials). The time needed to switch a dot of an LCD display from one luminance level to another is known as response time (RT). Instead of calculating the RT as the time needed to switch from full black to white as suggested by ISO 9241–305, most manufacturers provide the RT as the time needed to perform a grey-to-grey transition which is significantly shorter due to Response Time Compensation (RTC) mechanisms. RTC, also known as overdrive, is based on applying an over-voltage to accelerate the orientation change of the liquid crystals. This relation between luminance levels and response time in LCD displays hinders synchronous presentations of stimuli formed by different luminance levels [8].

Although there are significant differences between the underlying technologies of CRTs and LCDs, some mechanisms are similar [11] and they are operated in the same way from the operating system due to compatibility issues. Even in LCD monitors, the refresh rate configurations affect the monitor’s performance. In order to minimize flicker, tearing, and other artifacts, most programs use a technique called double-buffer (or multiple-buffer for cases with more than 2 buffers). While the graphics card is rastering one buffer on the screen, the software is preparing the next frame in the other buffer. At VSYNC signal, both buffers are flipped, and the process starts again. Therefore, different images cannot be shown on the screen at a rate faster than the refresh rate.

Because CRT monitors are decreasing in popularity with consumers, fewer are being produced, and it is becoming difficult for experimental laboratories to find CRT monitors through conventional supply channels. Some researchers fight this trend by buying and storing CRT monitors for future use or purchasing them through nonconventional supply channels (e.g., eBay) or even repairing them when they malfunction. However, these strategies do not seem viable in the long term. Therefore, we use a computer equipped with an LCD display to perform tests of software specialized for the presentation of visual stimuli.

### General Purpose and Real-time Operating Systems

To evaluate the influence of the real-time nature of an operating system in these experiments, some of the tests conducted in our study were run under a real-time operating system (Linux 2.6.33–29-realtime). It is important to recall how a real-time operating system differs from a general-purpose operating system. Contrary to popular belief, real-time operating systems are not necessarily faster, but they are predictable. Their schedulers work like traffic lights on a road. The schedulers’ main goal is not to maximize the overall throughput but, rather, to meet the predictability and fairness requirements. By contrast, the goal of general-purpose operating systems is usually the opposite. Throughput is maximized, even if background tasks are starved for long periods. As a result of their scheduling policies, the length of these periods is unpredictable. These non-real-time scheduling policies manage multitasking concurrency similar to a yield sign on a road (i.e., no task will wait unnecessarily if it can be attended to instantly, but the maximum running delay for a task when the system is overloaded is unpredictable). In general, a real-time and low-latency operating system is recommended for experiments that have high accuracy requirements. Unfortunately, nearly every specialized software package used in experimental psychology and neuroscience research runs on a general-purpose operating system (e.g., Microsoft Windows) and might be affected by the unpredictability of the task scheduling process. The good news is that specialized software eliminates eventual delays by implementing several optimizations (e.g., priority boosting and frame precomputation). However, optimizing twice (i.e., using a low-latency operating system and specialized software with built-in performance boosts) may lead to suboptimal resource allocation, which will result in poorer overall performance. Therefore, we tested whether the combination of a real-time operating system and the specialized software is optimal compared with the same software running on a general-purpose operating system. As will be explained further in the Results section, not all tests run using a real-time operating system were better than tests run using a general-purpose operating system in all conditions.

## Materials and Methods

We used Black Box Toolkit (BBTK) [12] to test the accuracy and precision of the timing mechanisms used to present visual stimuli by several specialized software packages on PCs: E-Prime 2.0.8.90 [13], DMDX 4.0.4.8 [14] and PsychoPy 1.64.00 [15]. All were tested on Microsoft Windows 7 32-bit Professional Edition. Because PsychoPy 1.64.00 can run natively on both operating systems, it was also tested on Ubuntu Linux 10.04 with the Linux 2.6.33-29-realtime kernel.

This subset of software packages is not aimed to be exhaustive but to serve as an example of the type of errors that can be found in their underlying timing mechanisms and the type of corrections that should be applied for time-sensitive experiments’ configurations. The selection of the investigated software packages has been done with the following criteria: select a popular commercial software package, a popular free software package, and a multiplatform software package. Regarding popularity, E-Prime is one of the most popular experimental software packages worldwide. Its combination of a graphical authoring tool and custom programming language to configure the experiments, its licensing model, and its underlying mechanisms to schedule and present stimuli make it a representative instance of this class (without detriment or other popular software packages such as DirectRT, NBS Presentation, etc.). DMDX is representative of a long tradition of experimental software, starting at 1975 with DMASTR and ending with its recent port to Microsoft DirectX libraries. In fact, these libraries are used by other software packages (e.g., NBS Presentation), therefore their limitations might affect all of them in a similar way. PsychoPy is a multiplatform software package that can run natively in Microsoft Windows, GNU/Linux and Apple Mac OS X. It is based on Python, like many other alternatives available (e.g., Experiment Builder, PyEPL, OpenSesame, Vision Egg), and provides a graphical authoring tool and a set of Python libraries to set up the experiments. All software tests were conducted using the same hardware: an Apple Macbook Pro with an Intel Core 2 Duo T7600 processor and an ATI Radeon Mobility X1600 graphics card. The native resolution of the display was 1440×900 pixels at 60 Hz. The suitability of the display was confirmed using the 6-hour *RefreshClockTest* of E-Prime (available at: http://www.pstnet.com/support/kb.asp?TopicID=3003) and an analysis of the results provided by the *timeByFrames* test of PsychoPy.

The BBTK photodetectors were used to measure the timing under all conditions. The BBTK was designed for this type of measurement [12] [16] and is able to transfer detected changes in luminance from the photodetector to the parallel port in less than 100 ns.

A well-known procedure was used to test the precision and accuracy of the visual stimuli presentations [17] [18]. For each of the experimental software packages examined, the photodetector, placed in the middle of the screen, was programmed to detect black to white and white to black screen transitions in the presentation display. In five independent series of 60 seconds, a continuous alternation of white and black screens was scheduled to be repeated with interval durations of 16.667, 50, 100, 200, 500, and 1000 ms (i.e., 1, 3, 6, 12, 30 and 60 ticks at 60 Hz, respectively). To compute the amount of missed frames, the whole set of measurements for each testing condition was used (i.e., approximately 300, 600, 1500, 3000, 6000, and 18000 for 1000, 500, 200, 100, 50, and 16.667 ms, respectively).

As in previous studies using this measurement procedure [17] [18], the presentation and measurement equipment used for all timing conditions are independent in order to avoid undesired interferences between the timing mechanisms used to generate the black to white and white to black transitions of the specialized software and the real-time application used to gather the data provided by the photodetector. The Apple MacBook Pro runs the experimental software independently (as it would be run in a real experiment with human participants), and the BBTK detects all changes in luminance from the Macbook’s display and sends them, via a parallel-port connection, to an auxiliary computer (AMD Sempron 2200 running the BBTK’s capture data software under Microsoft Windows XP 32-bit Professional edition).

## Results

**Figure 2:**
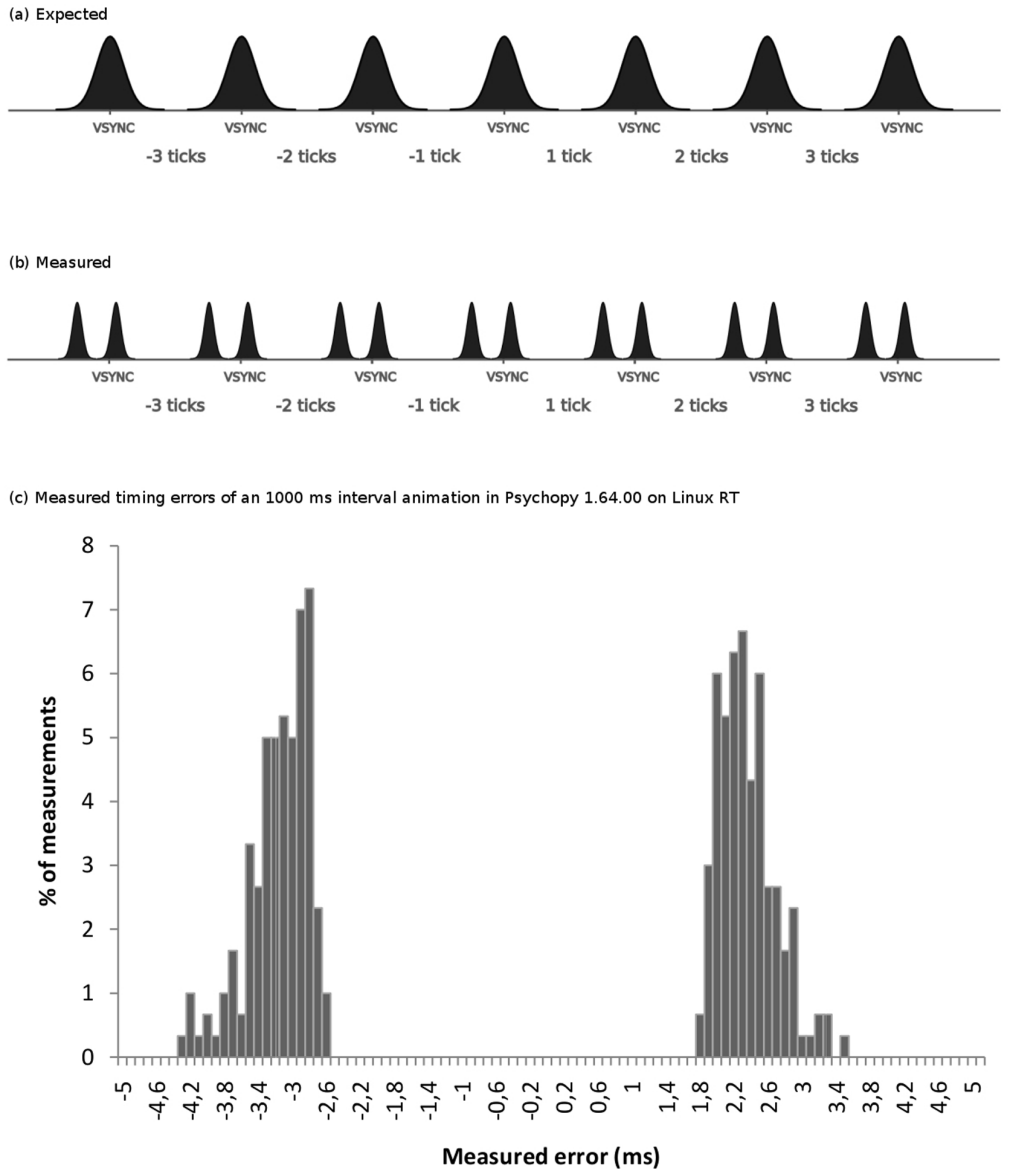
Measured timing errors distribution. Measured timing errors distribution: (a) Expected distribution of measured timing errors. (b) Actual distribution of measured timing errors. (c) Distribution of measured timing errors (in ms) of the PsychoPy 1.64.00 software displaying an animation from black to white every 1000 ms onLinux 2.6.33-29-realtime.

After considering all the previously mentioned details regarding displays, we expected that the difference between the interval configured by the experimenter for a visual stimulus presentation and the actual interval displayed by the software would be a multiple of the duration of a tick (see Figure 2a). However, we observed that all measured timing errors (MTEs) were concentrated around two peaks: one slightly before the VSYNC signal, the other slightly later. Figure 2b shows an ideal representation, and Figure 2c shows an example of the measurements collected by our photodetectors during the analysis of a 1000-ms interval animation performed using E-Prime.

The discrepancy between our expectations and the actual measurements can be explained by considering the rise and decay times of the LCD display. Because the response time is greater than 0 ms, these times are slightly longer for black-to-white transitions and slightly shorter for white-to-black transitions. Further technical details about rise and decay times of LCD displays and their implications in the duration of the stimuli are explained by Elze [8] [9] and Elze and Tanner [10]. Moreover, the BBTK photosensors do not provide a continuous analog value but a discrete digital one based on an adjustable threshold. This MTE is attributed to the technology used to show and measure the visual stimuli and not to the experimental software. Therefore, we decided to convert the MTE to full missed frames using the formula:
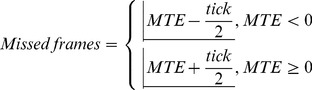
(1)


where 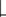 denotes the floor function.

Even with this conversion, we found discrepancies between stipulated and measured intervals in most of the setups we configured in DMDX, E-Prime and PsychoPy. These discrepancies were so consistent across all tests (each test was repeated 5 times per interval and software) that we decided to identify ways to compensate for them, as described below. We then repeated the measurements to assess the optimal precision and accuracy of the presentation of visual stimuli using the tested software. The results shown in Table 1 are the final measurements, once all adjustments and corrections had been made (the complete dataset can be found in https://openscienceframework.org/project/F2gBN.).

**Table 1:** Missed frames for each testing condition.

Software/OS	Interval(ms)	Expectancy(frames)	Missed frames					
			−2	−1	0	1	2	>2
DMDX	1000	300	0	0	300	0	0	0
/Windows 7	500	600	0	0	600	0	0	0
	200	1500	0	0	1500	0	0	0
	100	3000	0	0	2999	1	0	0
	50	6000	0	0	6000	0	0	0
	16.667	18000	0	0	17998	1	0	0
			**−2**	**−1**	**0**	**1**	**2**	**>2**
E-Prime	1000	300	0	0	299	1	0	0
/Windows 7	500	600	0	0	600	0	0	0
	200	1500	0	0	300	0	0	0
	100	3000	0	0	3000	0	0	0
	50	6000	0	0	5997	1	1	0
	16.667	18000	0	0	17857	28	20	12*
			**−2**	**−1**	**0**	**1**	**2**	**>2**
PsychoPy	1000	300	0	0	300	0	0	0
/Windows 7	500	600	0	0	600	0	0	0
	200	1500	0	2	1496	2	0	0
	100	3000	0	1179	634	1187	0	0
	50	6000	0	0	2572	2573	0	0
	16.667	18000	0	0	5981	5980	0	0
			**−2**	**−1**	**0**	**1**	**2**	**>2**
PsychoPy	1000	300	0	0	300	0	0	0
/Linux RT	500	600	0	9	582	9	0	0
	200	1500	2	13	1447	13	0	0
	100	3000	2	25	2889	32	2	0
	50	6000	0	0	5113	575	0	0
	16.667	18000	0	0	17908	14	3	10**

E-Prime, 16.667 ms interval, >2 missed frames distribution: 3 missed frames: 8; 4 missed frames: 3; 11 missed frames: 1.

PsychoPy under Linux RT, 16.667 ms interval, >2 missed frames distribution: 3 missed frames: 10.

In our initial DMDX configurations, we could not attain transition intervals shorter than three ticks (i.e., 50 ms at 60 Hz), even if the delay between trials was set to 0 ticks. In a personal communication, K. I. Forster confirmed that fast sequences of different items should not be presented as separate items but as a single item with multiple frames. This advice should be heeded by DMDX users when very short transitions between different trials are needed. Researchers should adjust related aspects of the experiment (e.g., non-visual stimuli presentations, reaction time measurement, or external apparatus synchronization) to compensate for this change.

We faced a different problem with E-Prime. Because durations in E-Prime are configured in milliseconds and not ticks, the duration of each interval was defined as a multiple of the number of milliseconds in one tick (i.e., 1000 ms for 60 ticks, 500 ms for 30 ticks, 200 ms for 12 ticks, etc.), and we requested VSYNC synchronization. After analyzing our initial measurements, we found that E-Prime was consistently missing a frame in each interval. Fortunately, information about these timing errors was provided in E-Prime’s log files, and they are related to the preparation time of the following stimulus (configured via the PreRelease value, which allows the current stimulus to release a portion of its execution time to a following stimulus in order to allow the following stimulus to perform setup activities) and E-Prime’s timing modes (i.e., Cumulative Mode and Event Mode). After testing several configurations, we concluded that the best way to optimize the precision and accuracy of visual stimuli presentations using E-Prime for this simple animation is to use E-Prime’s Event Mode timing (i.e., delays in the onset of an event will not affect the specified duration of the event), to subtract the duration of one tick from the interval duration in each transition (e.g., 1000 ms–16.667 ms = 983.333 ms) and to force the synchronization with VSYNC signal at the onset and offset of the stimulus. This adjustment is coherent with the “rule of thumb” provided by vendors in the user manual, according to which the stimulus duration should be set to 10ms below the expected total duration of the stimulus (“A good rule of thumb is to set the stimulus duration to 10 ms below the expected total duration of all refresh cycles desired for the stimulus. Since the visual duration is always rounded up to the next refresh, the display duration must be specified as some portion of one refresh below the targeted refresh duration plus the expected measurement error for when to look for the vertical blanking signal indicating the time of a refresh.” [13]). But instead of using an arbitrary correction value, we decided to follow a more detailed suggestion provided in the E-Prime users’ manual and make this correction value dependent on the refresh rate of the monitor and the time needed to prepare the next frame of the animation (i.e., a non-zero but negligible value in our simple animation).

Our tests using PsychoPy also required adjustments. We found that a black frame lasting 1 tick was introduced in every frame transition, which increased the duration of the black intervals by two ticks (one before the beginning of the interval and another after the end) and had no effect in the duration of white intervals. To compensate for this, we subtracted two ticks from the duration of the black intervals (e.g., 1000 ms–2×16.667 ms = 966.667 ms). For intervals lasting 1 tick, a continuous white to white transition 1 tick in length was configured because PsychoPy automatically inserted one black frame 1 tick in duration between them.

**Figure 3:**
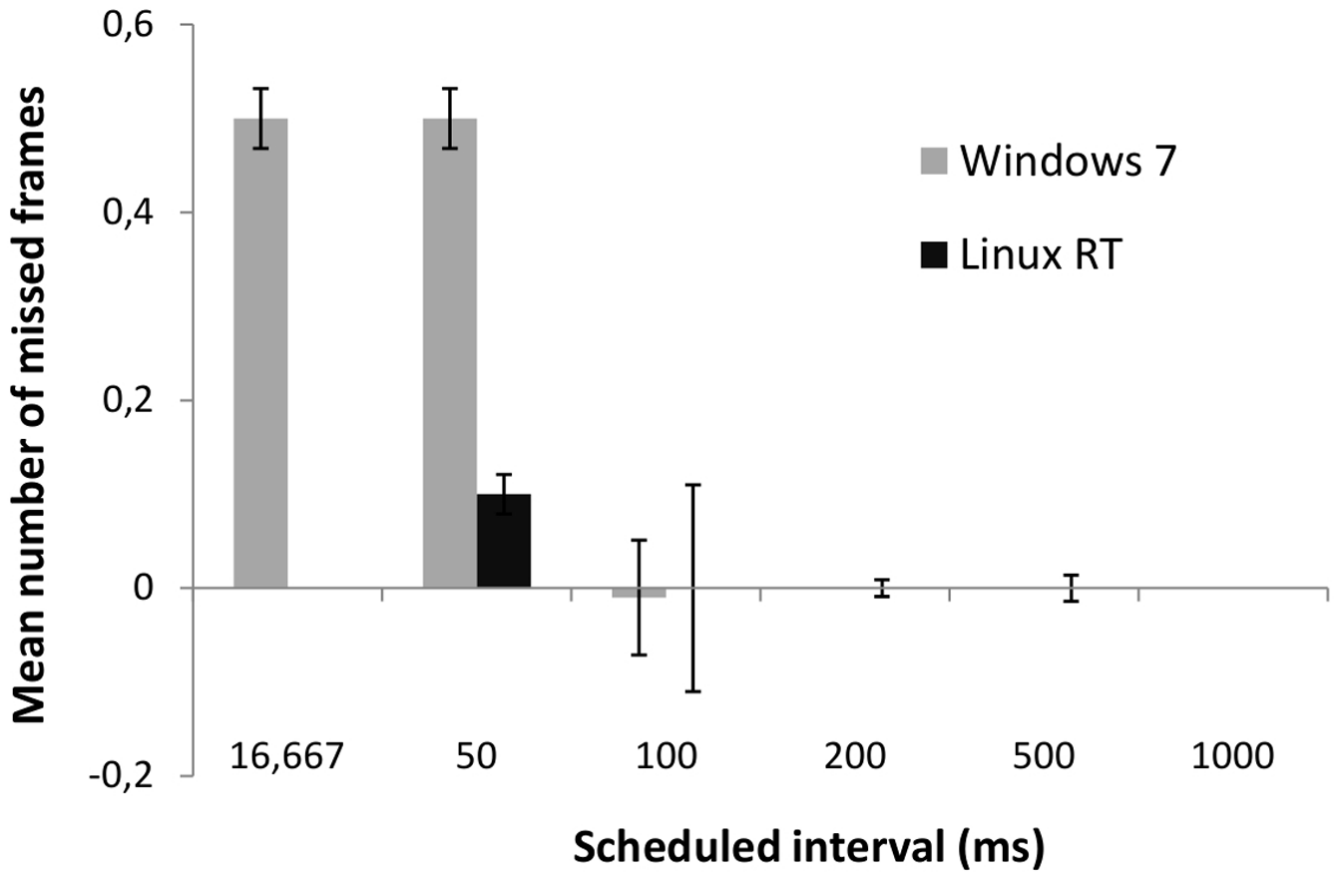
Absolute number of missed frames in PsychoPy tests. Absolute number of missed frames for each testing condition of PsychoPy running on Microsoft Windows 7 Professional 32-bit edition and Ubuntu 10.04 LTS with Linux 2.6.33–29-realtime.

As mentioned previously, the results shown in Table 1 represent the missed frames (i.e., the number of frames away from the target) for intervals lasting 1000, 500, 200, 100, 50, and 16.667 ms, respectively once all adjustments were performed. Note that the number of missed frames in each row do not necessarily add up to the expected number of frames. This happens because exactly 5 minutes of black-to-white and white-to-black transitions were recorded. If any of the frames happened to be presented later than scheduled, then the 5 minute-interval might not suffice to present the whole sequence of programmed transitions. Positive values represent intervals longer than the configured interval, negative values represent shorter intervals, and zero means the interval lasted the stipulated duration. Quite interestingly, the number of missed frames is noticeably higher when the shortest intervals were tested with PsychoPy, both in Microsoft Windows and Linux. Figure 3 depicts the mean number of missed frames when using PsychoPy under both OS. To improve the comparability across time intervals, we computed these means using only the first 50 transitions in each round (250 transitions in each condition). As can be seen, the performance of PsychoPy is worse under very brief intervals, but this effect is somewhat ameliorated when PsychoPy operates under realtime-Linux.

## Discussion

In all of our tests, DMDX presented visual stimuli with high precision and accuracy. Were it not for the difficulty in programming experiments using the DMASTR syntax, it would be a perfect tool. DMDX allows defining time intervals both in ticks and in milliseconds. Although defining stimuli durations in ticks can be tricky, it is closer to the actual implementation of the experiment and makes the limitations of the hardware explicit to researchers, which discourages them from setting intervals that are impossible to meet (i.e., those that are not a multiple of the tick duration). In fact, as Forster explains in the DMDX Online Help page [19], millisecond-oriented keywords are only provided for one of two situations: (1) when researchers do not care about precise tachistoscopic presentations, or (2) when the item file is needed to work on multiple machines regardless of their refresh rates. Moreover, very short stimuli presentations have to be aggregated as different frames of the same trial to eliminate inter-trial delays. This change can interfere with other aspects of the experimental design (e.g., non-visual stimuli presentations, reaction time measurements, etc.) or be overlooked by experimenters.

We conclude that E-Prime is a highly precise software package for the presentation of visual stimuli. However, setting the stimuli durations in milliseconds (and not in ticks) might introduce a source of error. On the one hand, it is much easier for researchers to use. They are accustomed to thinking in terms of seconds or milliseconds and may not be aware of the concept of a tick. On the other hand, using presentation times that are not multiple of the refresh rate may lead to timing errors. These errors can be smaller than the duration of one tick, but will still increase the measured error. According to our tests, even visual stimuli with a duration that is a multiple of the refresh rate may have to wait to be synchronized with the VSYNC signal of the display. This delay results in larger presentation times for the previous stimulus. As mentioned previously, correcting this deviation is straightforward. However, if a researcher focuses on the reaction times in the E-Prime logs and does not analyze the registered timing errors during the presentation of stimuli, this error can be overlooked. We recommend that researchers set the VSYNC signal synchronization and subtract the duration of one tick when defining stimuli intervals, and ensure that the pre-computation time needed to prepare stimuli does not exceed on that subtracted tick plus the amount of time configured in the PreRelease value of the stimulus.

Both PsychoPy and E-Prime provide the experimenter with a user-friendly interface for setting up experiments and the ability to define arbitrary times for the duration of the stimuli. Therefore, similar recommendations apply. In addition, PsychoPy automatically includes a black frame lasting 1 tick in every stimuli transition. This inclusion should be taken into account in experimental paradigms with high precision and accuracy requirements, and the frame duration should be corrected by subtracting the black frames when possible. Moreover, PsychoPy is able to eliminate the limitations of the Graphical User Interface (GUI) by providing an Application Programming Interface (API) from which experiments can be easily developed in Python. Combining both high-level (i.e., GUI) and low-level (i.e., Python API) approaches, experimenters are able to generate experimental setups that are as accurate as the underlying platform (e.g., using frame refresh periods to control presentation timing accurately). Therefore, PsychoPy is an attractive alternative for designing and running cognitive experiments with some very interesting characteristics. For example, it is published under a Free Software license. In addition, it runs under the most popular general-purpose operating systems (i.e., Microsoft Windows, GNU/Linux and Mac OS X) and is even able to take advantage of the reliability provided by real-time operating systems (e.g., Linux 2.6.33–29-realtime).

The significant differences between a multi-platform software package such as PsychoPy and single-platform alternatives like E-Prime or DMDX are related to the software abstraction layers involved in each case. DMDX uses a multimedia library specifically designed for the development of real-time applications (i.e., DirectX) and requires pre-configuration of display equipment to achieve the maximum accuracy and precision. E-Prime is not as close to the hardware as DMDX, but leverages best timing APIs in Microsoft Windows (i.e., QueryPerformanceCounters and Multimedia Timers) to maximize the accuracy and precision of stimulus presentations. PsychoPy, by contrast, relies on a high-level and multi-platform interpreted language (i.e., Python). The benefits of this approach are clear: any PsychoPy-based experiment can run smoothly on all major operating systems. However, the transition from PsychoPy experiment setup files (i.e., XML-based psyexp files) to the control of the display hardware might be performed sub-optimally if not tuned manually. The good news is that PsychoPy gives advanced users the ability to maximize the accuracy and precision of stimulus presentation when used as a Python library through the use of a non-slip (global) clock timing mechanism. This functionality is also available from the Experiment Builder of PsychoPy since version 1.74.00. Moreover, Python scripts generated by PsychoPy could improve their performance if compiled to native executable code or used an optimized Python interpreter.

There may be several causes that explain the differences between the results gathered using PsychoPy on Microsoft Windows and on Linux real-time. The best-effort policy of a non-real-time operating system like Microsoft Windows is able to provide a better throughput than a real-time operating system in non demanding cases (i.e., tests with larger intervals), while performs worse in demanding situations (i.e., tests with shorter intervals). Moreover, the software architecture of each operating system is significantly different regarding the graphical layer (i.e., it runs as a user process in Linux, whereas it is integrated in the kernel in Microsoft Windows), and might be the cause of a slightly poorer performance in Linux in tests where the stress of the system is not an issue.

Further research is needed to extend these conclusions to a wider range of experimental software. Providing sub-millisecond accuracy is a typical claim of vision-related experimental software, but the validity of these claims should be tested by third-party researchers. Nevertheless, although some parameters (e.g., the refresh rate of the LCD display) were not optimal, the overall performance of the tested operating systems and experimental software combinations were sufficient to fulfill the requirements of most of the experimental tasks performed by researchers. This is good news for researchers who need to present stimuli with high accuracy. Provided that the recommendations explained in the present work are taken into account by researchers, we consider DMDX, E-Prime and PsychoPy suitable for most of the experimental paradigms used in cognitive research that require high levels of precision and accuracy.
